# Ornithine cyclodeaminase-based proline production by *Corynebacterium glutamicum*

**DOI:** 10.1186/1475-2859-12-63

**Published:** 2013-06-28

**Authors:** Jaide Vold Korgaard Jensen, Volker Fritz Wendisch

**Affiliations:** 1 Genetics of Prokaryotes, Faculty of Biology & CeBiTec, University of Bielefeld, Universitätsstrasse 25, 33615, Bielefeld, Germany

**Keywords:** Amino Acid, Proline, *Corynebacterium Glutamicum*, Metabolic Engineering, OCD, ORN1, Ornithine, Ornithine Cyclodeaminase, Platform Strain, Diamine, Putrescine, N-acetylglutamate Kinase

## Abstract

**Background:**

The soil bacterium *Corynebacterium glutamicum*, best known for its glutamate producing ability, is suitable as a producer of a variety of bioproducts. Glutamate is the precursor of the amino acid proline. Proline biosynthesis typically involves three enzymes and a spontaneous cyclisation reaction. Alternatively, proline can be synthesised from ornithine, an intermediate of arginine biosynthesis. The direct conversion of ornithine to proline is catalysed by ornithine cyclodeaminase. An ornithine overproducing platform strain with deletions of *argR* and *argF* (ORN1) has been employed for production of derived compounds such as putrescine. By heterologous expression of *ocd* this platform strain can be engineered further for proline production.

**Results:**

Plasmid-based expression of *ocd* encoding the putative ornithine cyclodeaminase of *C. glutamicum* did not result in detectable proline accumulation in the culture medium. However, plasmid-based expression of *ocd* from *Pseudomonas putida* resulted in proline production with yields up to 0.31 ± 0.01 g proline/g glucose. Overexpression of the gene encoding a feedback-alleviated N-acetylglutamate kinase further increased proline production to 0.36 ± 0.01 g/g. In addition, feedback-alleviation of N-acetylglutamate kinase entailed growth-coupled production of proline and reduced the accumulation of by-products in the culture medium.

**Conclusions:**

The product spectrum of the platform strain *C. glutamicum* ORN1 was expanded to include the amino acid L-proline. Upon further development of the ornithine overproducing platform strain, industrial production of amino acids of the glutamate family and derived bioproducts such as diamines might become within reach.

## Background

The workhorse *Corynebacterium glutamicum* has for decades been used as an amino acid producer. Although, in terms of quantity, the main contributors to the amino acid market are L-lysine and L-glutamate, minor amino acids such as L-proline are also of importance. Proline is predominantly used as an organocatalyst by the chemical industry, as a precursor for compounds with pharmaceutical and cosmetic applications, and as a feed additive [1-3]. The natural functions of proline in prokaryotic and eukaryotic cells have been reported to be as an osmolyte, a potential virulence factor for some pathogenic bacteria, and a source of carbon, nitrogen, and energy [4]. The amino acid functions as a compatible solute of *C. glutamicum* and in this respect the organism has been shown to grow at intracellular concentrations of up to 94 g/L proline with no determined upper limit [5]. In *Escherichia coli* the bifunctional enzyme PutA catalyses the two-step oxidation of proline to glutamate with proline dehydrogenase and Δ1-pyrroline-5-carboxylate dehydrogenase activities: reactions that occur at high proline concentrations [6]. *C. glutamicum* contains a putative *putA* gene, but the activity of the encoded enzyme has thus far not been confirmed. It is not clear whether *C. glutamicum* can utilise proline as a carbon or nitrogen source as contradictory statements about proline utilisation by this bacterium have been published [7,8].

**Figure 1:**
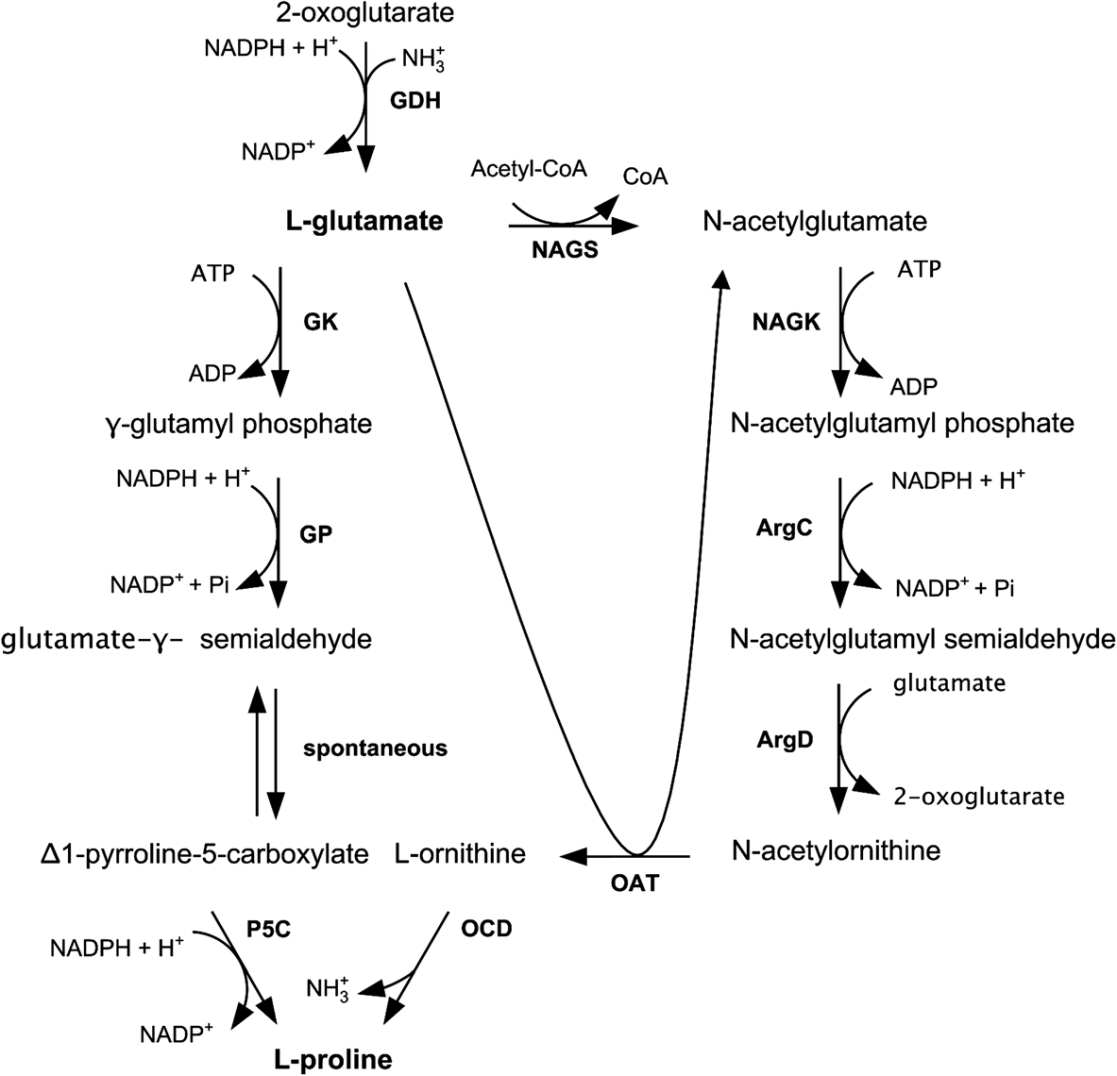
**Proline biosynthesis in *Corynebacterium glutamicum*.** Reactions and enzymes of the proline and ornithine biosynthetic pathways of *Corynebacterium glutamicum* are depicted together with the reaction catalysed by ornithine cyclodeaminase (OCD): glutamate dehydrogenase (GDH) encoded by *gdh*, γ-glutamyl kinase (GK) encoded by *proB*, γ-glutamyl phosphate reductase (GP) encoded by *proA*, pyrroline-5-carboxylate reductase (P5C) encoded by *proC*, N‒acetylglutamate synthase (NAGS) activity, N‒acetylglutamate kinase (NAGK) encoded by *argB*, N‒acetyl‒γ‒glutamyl‒phosphate reductase (ArgC) encoded by *argC*, acetylornithine aminotransferase (ArgD) encoded by *argD*, ornithine acetyltransferase (OAT) encoded by *argJ*, and OCD encoded by *ocd*.

Proline is synthesised from glutamate via three enzymatic and one spontaneous reactions, in most investigated microorganisms [4,9,10]. The enzymes of the proline pathway encoded by *proB, proA,* and *proC* catalyse the phosphorylation of glutamate followed by reduction to glutamate-γ-semialdehyde, a spontaneous cyclisation, and finally the reduction to proline. An alternative route to proline biosynthesis involves ornithine cyclodeaminase (OCD) which catalyses the conversion of ornithine to proline and ammonia with deamination of the α-amino group prior to cyclisation (Figure 1). However, only a few organisms such as *Clostridium sporogenes*, *Treponema denticola*, *Agrobacterium tumefaciens*, and *Pseudomonas putida* have been reported to contain OCD [11-15]. The genome of *C. glutamicum* contains a putative *ocd* gene, however, evidence for its function as ornithine cyclodeaminase has not been reported [16].

In *C. glutamicum* ornithine is an intermediate of arginine biosynthesis and is synthesised from glutamate through five enzymatic steps where the second enzyme, N-acetylglutamate kinase (NAGK), is feedback-inhibited by arginine [17]. It is known that the arginine biosynthetic pathway of *C. glutamicum* is regulated at the transcriptional level by the repressor ArgR that has been shown to bind upstream regions of *argC*, *argB*, *argF*, and *argG*[18]. Further genetic regulation, although not fully comprehended, is concerted by the acyl-responsive transcriptional regulator FarR and by the potential allosteric inhibition of ornithine acetyltransferase by ornithine [18,19].

An ornithine overproducing *C. glutamicum* strain [20,21] with deletions of the genes *argR* and of *argF*, the gene encoding ornithine carbamoyl-transferase, which converts ornithine to citrulline in the arginine biosynthetic pathway, has been constructed. This strain, called ORN1, has the potential of serving as a platform for the production of several industrially relevant bioproducts of the glutamate family; namely ornithine, citrulline, arginine, putrescine, and spermidine. Based on *C. glutamicum* ORN1, strains PUT1 [22] and PUT21 [23] have been developed for production of 1,4-diaminobutane (putrescine), and ARG1 [20] is an arginine-producing ORN1 derivative. Here, the production of proline based on ORN1 is reported. Heterologously produced ornithine cyclodeaminase from *P. putida* led to a conversion of ornithine to proline and thereby constitutes an expansion of the product palette of the platform strain ORN1.

## Results

### Proline utilisation

To establish if the putative proline degradation system of *C. glutamicum* had an effect on extracellular accumulation of proline, we first investigated the utilisation of proline as carbon and nitrogen source. Wild-type *C. glutamicum* was inoculated to an optical density (OD) of 1 in CGXII minimal medium with 20 g/L glucose or a C-equimolar concentration of proline as carbon source and 20 g/L ammonium sulfate and 5 g/L urea or a N-equimolar concentration of proline as nitrogen source. For cells cultured in medium with proline as carbon source no biomass formation was observed during 48 h of incubation. An OD of ~30 could be reached within 10 h by cultivation in CGXII medium with glucose, ammonium sulfate, and urea. When an N-equimalor proline concentration was used as the sole nitrogen source, the cells were able to duplicate twice in 24 h within the 48 h of incubation resulting in a maximum OD of ~3.5. For comparison an OD of ~1.5 could be reached when no nitrogen source was added to the medium.

### Plasmid-based overexpression of *ocd*

**Figure 2:**
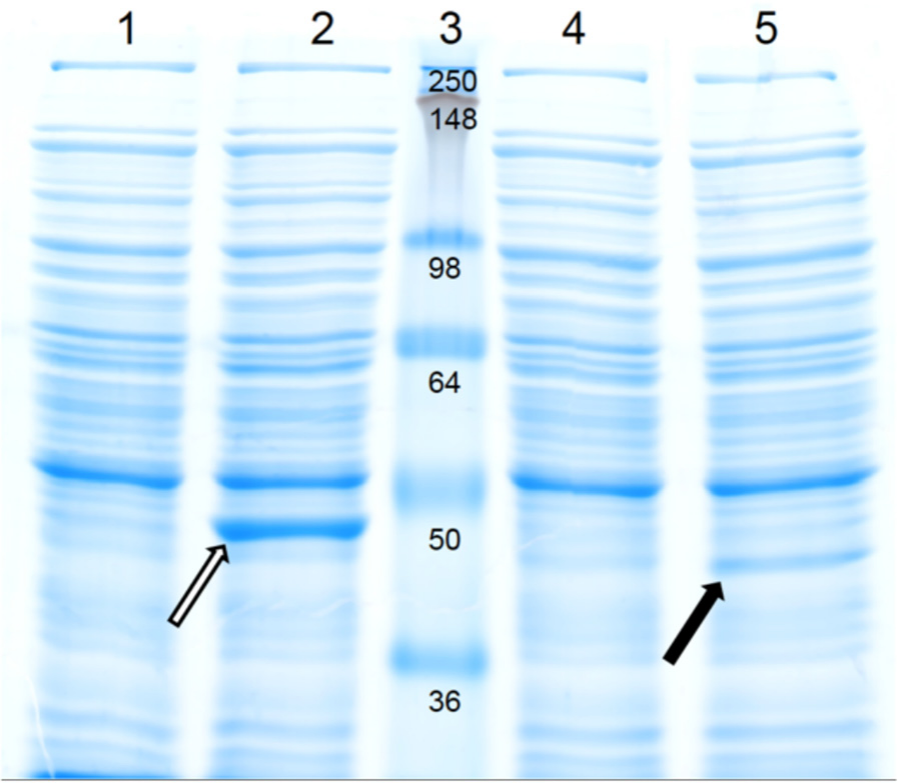
**SDS-PAGE analysis of crude extracts of *C. glutamicum *strains JJ001, JJ002, JJ003, and JJ004.** JJ001 (lane1), JJ002 (lane 2), protein standard (lane 3), JJ003 (lane 4), and JJ004 (lane 5). The deduced molecular mass of putative OCD from *Corynebacterium glutamicum* in JJ002 is 41 kDa (open arrow) and OCD from *Pseudomonas putida* in JJ003 and JJ004 is 38 kDa (filled arrow). The units of the protein standard are in kDa. Crude extracts were prepared from cells grown in BHI with 1 mM IPTG and 25 μg/mL kanamycin.

The gene encoding the biochemically characterised OCD from *P. putida*[24] and the putative *ocd* from *C. glutamicum* were cloned into the IPTG-inducible expression plasmid pVWEx1 [25]. The resulting plasmids pVWEx1-*ocd*_*Cg*_ and pVWEx1-*ocd*_*Pp*_ were transformed into the ornithine producer ORN1 to yield strains JJ002 (ORN1 carrying pVWEx1-*ocd*_*Cg*_) and JJ003 (ORN1 carrying pVWEx1-*ocd*_*Pp*1_). For a presumed more efficient translational termination, the original stop codon TGA of *ocd*_*Pp*_ was replaced by the more frequently used TAA (JJ004). The specific cyclodeaminase activities in crude extracts of these strains and the control strain JJ001 (ORN1 carrying pVWEx1) were determined and the presence of the overproduced proteins visualised by SDS-PAGE (Figure 2, Table 1). No activity could be observed for crude extracts of JJ001 and JJ002, although the crude extract of JJ002 exhibited a band on an SDS-gel corresponding to the weight of the putative *C. glutamicum* OCD of 40.96 kDa, calculated based on the amino acid sequence (Figure 2). Substitution of the translational stop codon caused an almost 12-fold increased specific activity of OCD_*Pp*_.

**Table 1:** Ornithine cyclodeaminase activity

**Crude extract**	**Specific activity**
	**(μmol/min/mg protein)**
JJ001	< 4·10^-4^
JJ002	< 4·10^-4^
JJ003	0.06 ± 0.02
JJ004	0.71 ± 0.09

Crude extracts were obtained by sonication of cells cultured in BHI medium supplemented with 1 mM IPTG and 25 μg/mL kanamycin.

**Figure 3:**
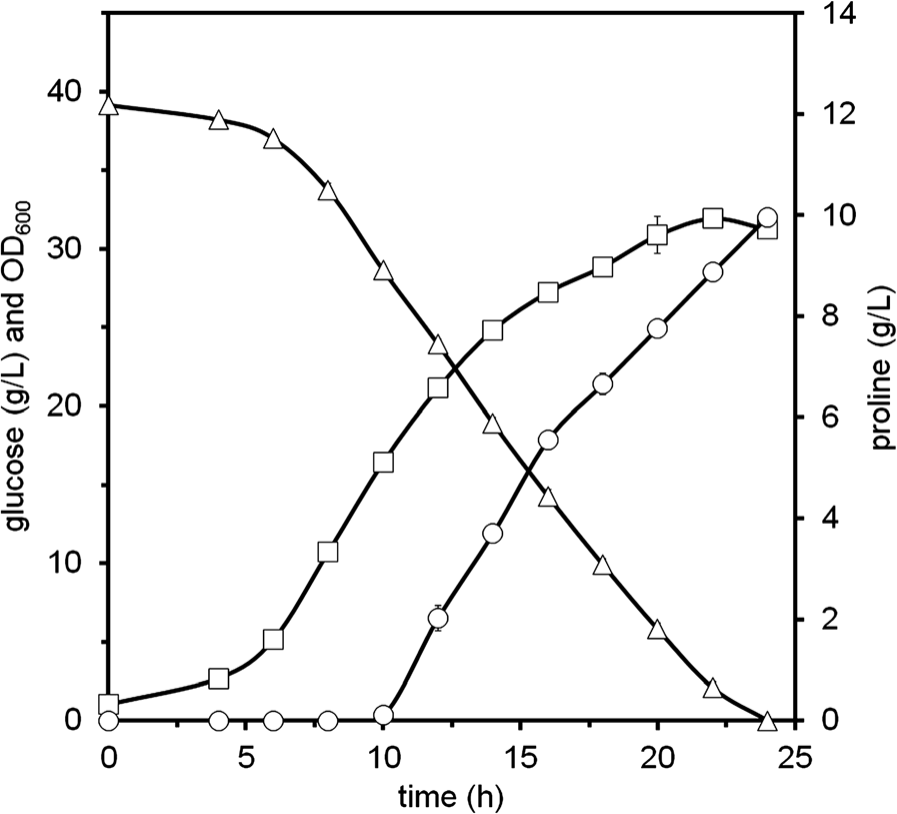
**Growth, substrate consumption, and proline formation by *C. glutamicum *JJ004 in CGXII minimal medium containing 40 g/L glucose, 0.75 mM arginine, 1 mM IPTG, and 25 μg/mL kanamycin.** Open square, biomass; open triangle, glucose; open circle, proline. Data are means and standard deviations of at least three cultivations.

Shake flask fermentations in glucose minimal medium with IPTG were performed with the aforementioned strains. Samples were withdrawn for product quantification by HPLC. Upon glucose depletion, proline could not be detected in the supernatants of strains JJ001 and JJ002, whereas ornithine was produced by both strains (Table 2). By contrast, JJ003 and JJ004 accumulated proline in the supernatant (Figure 3, Table 2). Owing to the feedback inhibition of NAGK by arginine, production of proline was growth decoupled. Strain JJ004 accumulated about three folds more proline than JJ003 indicating that the improved translational termination of *ocd*_*Pp*_ entailed not only increased OCD activity, but also increased proline production. All strains accumulated ornithine and the by-products threonine, alanine, and valine. Furthermore, trace amounts of glutamate (up to 5 μm) could be detected, except for strain JJ003 where 0.24 ± 0.1 g/L was accumulated.

**Table 2:** Accumulation of proline and by-products

	**Proline (g/L)**	**Ornithine (g/L)**	**Threonine (g/L)**	**Alanine (g/L)**	**Valine (g/L)**
JJ001	< 10^-4^	14.8 ± 0.2	0.3 ± 0.01	0.7 ± 0.03	0.7 ± 0.01
JJ002	< 10^-4^	12.1 ± 0.3	0.3 ± 0.01	0.5 ± 0.01	0.5 ± 0.01
JJ003	2.4 ± 0.1	5.5 ± 0.3	0.3 ± 0.01	0.6 ± 0.01	0.4 ± 0.01
JJ004	10.0 ± 0.1	2.8 ± 0.2	0.2 ± 0.01	0.3 ± 0.01	0.3 ± 0.01
JJ004*	10.9 ± 0.3	0.2 ± 0.1	0.2 ± 0.01	0.3 ± 0.01	0.6 ± 0.01

Titers were obtained from cells grown in CGXII medium with 40 g/L glucose, 0.75 mM arginine, 1 mM IPTG, and 25 μg/mL kanamycin. * Titers were obtained from cells grown in CGXIIm medium with 35 g/L glucose and supplements as stated above.

### Identification of key medium components for proline production

As deamination of ornithine by OCD yields ammonia besides proline, and as CGXII contains a high nitrogen concentration, it was tested if the nitrogen content of the medium had an effect on proline formation and if other medium components influenced production. First the effect of the nature of the nitrogen source was tested; here urea, and/or ammonium sulphate or ammonium chloride were selected (Table 3). Urea as sole nitrogen source at the concentration tested appeared to be superior for proline overproduction.

**Table 3:** Proline production with different nitrogen sources

**Nitrogen source**	**Proline (g/L)**
Urea, (NH_4_)_2_SO_4_	8.0 ± 0.2
Urea	11.5 ± 0.4
(NH_4_)_2_SO_4_	6.2 ± 0.1
NH_4_Cl	6.0 ± 0.1

Titers were obtained from cells grown in CGXII medium with 40 g/L glucose, 0.75 mM arginine, 1 mM IPTG, and 25 μg/mL kanamycin.

**Figure 4:**
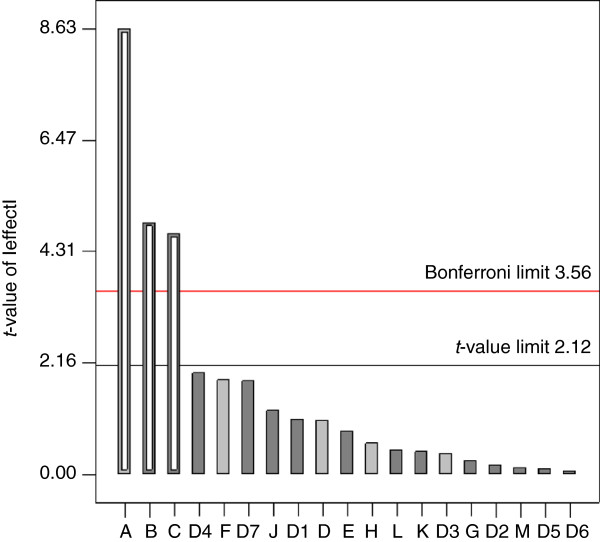
**Pareto chart showing the standardised effects of medium components on proline production.** Light grey, the influence of the tested factor upon proline production is greater at the high concentration; dark grey, the influence of the tested factor upon proline production is greater at the low concentration. Statistically significant effects (open columns) are differentiated from insignificant effects (closed columns). A-L and D1-D7 are defined in Table 6.

After the initial screen a Plackett-Burman design was used to identify key components in the medium affecting proline production and to verify that the nitrogen source significantly affects proline production. The design made it possible to determine the relevant factors with a small number of trials. Twelve factors were screened; all components of CGXII medium and in addition IPTG and arginine. The experimental design and responses are shown in Table 4. Significant effects on proline production were observed when concentrations of glucose, urea, and monopotassium phosphate were varied (Figure 4). A positive effect on proline production was observed for high concentrations of glucose, while low concentrations of urea and monopotassium phosphate improved proline production. As the *t*-values of the effects of glucose, urea, and monopotassium phosphate lie above not only the *t*-value threshold, but also the conservative Bonferroni threshold, the components are more likely to be key medium components and their effect not stochastic.

**Table 4:** Plackett-Burman design represented by coded values, and proline concentration as the response

**Run**	**A**	**B**	**C**	**D**	**E**	**F**	**G**	**H**	**I**	**J**	**K**	**L**	**D1**	**D2**	**D3**	**D4**	**D5**	**D6**	**D7**	**Response**
																				**Proline (g/L)**
1	-	-	+	+	+	+	-	+	-	+	-	-	-	-	+	+	+	+	+	4.3 ± 0.01
2	-	+	-	-	-	-	+	+	-	+	+	-	-	+	+	+	+	-	+	3.4 ± 0.05
3	+	+	+	-	+	-	+	-	-	-	-	+	_+_	-	+	+	+	-	+	4.1 ± 0.4
4	+	-	+	-	+	-	-	-	-	+	+	-	+	+	-	-	-	+	+	5.8 ± 0.2
5	+	-	-	+	+	+	+	-	+	-	+	-	-	-	-	+	+	-	+	7.9 ± 0.3
6	+	-	-	-	-	+	+	-	+	+	-	-	+	+	+	+	+	+	-	8.2 ± 0.08
7	-	-	-	-	+	+	-	+	+	-	-	+	+	+	+	-	-	-	+	5.3 ± 0.1
8	-	-	+	+	-	+	+	-	-	+	+	+	+	-	+	-	-	-	-	4.9 ± 0.01
9	+	-	+	+	-	-	+	+	+	+	-	+	-	+	-	-	-	-	+	6.7 ± 0.1
10	+	+	+	+	-	+	-	+	-	-	-	-	+	+	-	+	+	-	-	5.9 ± 0.05
11	+	+	-	+	+	-	-	+	+	+	+	-	+	-	+	-	-	-	-	6.8 ± 0.08
12	+	+	-	-	+	+	+	+	-	+	-	+	-	-	-	-	-	+	-	7.7 ± 0.4
13	-	-	-	-	-	-	-	-	-	-	-	-	-	-	-	-	-	-	-	6.2 ± 0
14	+	+	-	+	-	+	-	-	-	-	+	+	-	+	+	-	-	+	+	7.8 ± 0.4
15	-	+	+	+	+	-	+	-	+	-	-	-	-	+	+	-	-	+	-	3.0 ± 0.2
16	+	-	+	-	-	-	-	+	+	-	+	+	-	-	+	+	+	+	-	6.5 ± 0.2
17	-	+	+	-	+	+	-	-	+	+	+	+	-	+	-	+	+	-	-	2.5 ± 0.2
18	-	+	-	+	-	-	-	-	+	+	-	+	+	-	-	+	+	+	+	3.0 ± 0.2
19	-	+	+	-	-	+	+	+	+	-	+	-	+	-	-	-	-	+	+	2.7 ± 0.07
20	-	-	-	+	+	-	+	+	-	-	+	+	+	+	-	+	+	+	-	5.1 ± 0.09

+ indicates that the medium component was used at its high level concentration.

– indicates that the medium component was used at its low level concentration.

Thus, three different concentrations of urea (2.5, 5 and 7.5 g/L), glucose (20, 35, and 50 g/L) and monopotassium phosphate (0.5, 1.25, and 2 g/L) were tested (data not shown) and an improved medium was derived (5 g/L urea as nitrogen source, 35 g/L glucose as carbon source and 2 g/L potassium phosphate) and was used for further proline production experiments. Fermentations of JJ004 in the modified medium increased proline production by 25% compared to production in CGXII medium (0.31 ± 0.01 as compared to 0.25 ± 0.003 g proline/ g glucose, Table 2) and ornithine accumulation was reduced.

### Coupling growth to proline production

**Figure 5:**
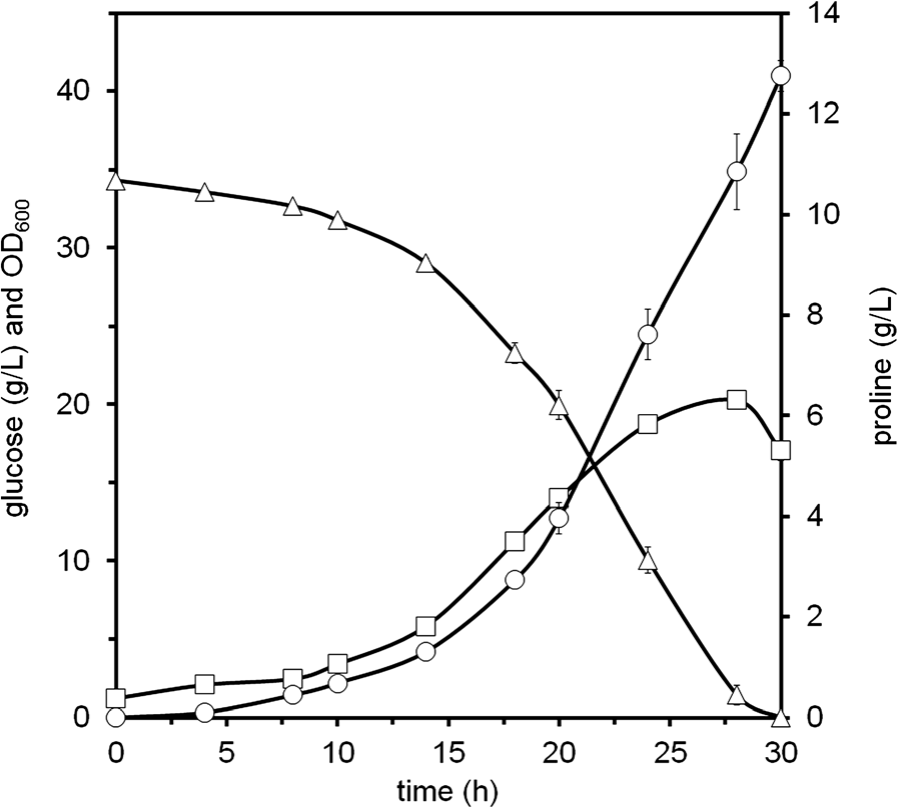
**Growth, substrate consumption, and proline formation by *C. glutamicum *JJ006 in modified CGXII medium containing 35 g/L glucose, 0.75 mM arginine, 1 mM IPTG, and 25 μg/mL kanamycin.** Open square, biomass; open triangle, glucose; open circle, proline. Data are means and standard deviations of at least three cultivations.

In *C. glutamicum* the second enzyme of the ornithine pathway NAGK is feedback inhibited by arginine. The feedback inhibition therefore constitutes a rate-limiting step at high arginine concentrations. Hence, both the feedback alleviation of NAGK and leaky expression of *argF* have been employed as means to increase/ couple growth to production [20,23]. Therefore, a feedback alleviated NAGK was overproduced in JJ004 and the resulting strain JJ006 was assayed for NAGK activity. After induction by 1 mM IPTG and growth in BHI complex medium crude extracts of strain JJ006 showed 94 ± 7 mU/mg specific NAGK activity, which was three fold higher than in the respective control stain JJ005 (30 ± 7 mU/mg; data not shown). Overproduction of feedback alleviated NAGK entailed increased proline production and reduced by-product formation (Figure 5, Table 5). Less biomass was formed and the growth rate was reduced, however, proline production started early during growth.

**Table 5:** Accumulation of proline and byproducts

	**Proline (g/L)**	**Ornithine (g/L)**	**Threonine (g/L)**	**Alanine (g/L)**	**Valine (g/L)**
JJ005	11.7 ± 0.1	< 10^-4^	0.2 ± 0.005	0.09 ± 0.005	0.28 ± 0.004
JJ006	12.7 ± 0.3	< 10^-4^	0.09 ± 0.003	0.09 ± 0.002	0.16 ± 0.001

Titers were obtained from cells grown in CGXIIm with 35 g/L glucose, 0.75 mM arginine, 1 mM IPTG, 25 μg/mL kanamycin, and 50 μg/mL spectinomycin.

## Discussion

*C. glutamicum* is, especially with regards to carbon and amino acid metabolism, a well-studied bacterium. Nevertheless, details on the proline degradation pathway and regulation of the proline and arginine biosynthetic pathways remain to be elucidated. *C. glutamicum* can utilise several amino acids as sole carbon and/or nitrogen source [7] e.g. glutamine has been demonstrated to be an excellent nitrogen source and also allows growth when used as sole carbon and nitrogen source [26]. In the case of proline contradictory observations of its utilisation as nitrogen source have been made [7,8]. Bott & Niebisch reported that *C. glutamicum* could utilize proline as carbon and nitrogen source, however very slowly. In this study, it was shown that proline did not serve as sole source of carbon for growth of *C. glutamicum*, and that it is a very poor source of nitrogen.

When comparing proline synthesis starting from 2-oxoglutarate via the proline pathway to the conversion via the ornithine pathway, there is a difference in the requirement for ammonia. In the proline biosynthetic pathway one molecule of ammonium is assimilated in the reductive amination of 2-oxoglutarate to glutamate by glutamate dehydrogenase (GDH) per molecule of proline produced (Figure 1). In the OCD-based pathway, a second ammonium molecule is required to be assimilated by GDH, however, it is released during the final ornithine cyclodeaminase reaction. Therefore the conversion of 2-oxoglutarate to glutamate via GDH must be twice as high in the OCD-based pathway as compared to the proline pathway. By medium optimisation employing the Plackett-Burman design it could be shown that a low urea concentration had a positive effect on proline accumulation. While urea utilisation needs to be induced, ammonium assimilation via GDH only shows a weak dependency on nitrogen availability [27]. Besides the low affinity assimilation to ammonium via GDH, *C. glutamicum* also possesses the high affinity GS-GOGAT system for ammonium assimilation which is induced upon nitrogen starvation [27]. The *in vivo* fluxes of ammonia assimilation via GDH and GS-GOGAT could be determined in *C. glutamicum* ATCC 13032 and a direct dependency of flux via the GS-GOGAT system on ammonium availability was observed [27]. Under C-limited conditions in a continous culture GS and GOGAT activities in crude extracts were significantly reduced [27]. Proline production was performed with sufficient ammonium, therefore it is assumed that GDH primarily contributes to ammonium assimilation under these conditions.

The genome of *C. glutamicum* contains a gene annotated to encode ornithine cyclodeaminase [16] within the nitrogen-regulated putative *amt-ocd-sox* operon [28]. Overexpression of this operon was beneficial for lysine production, but the molecular mechanism remained unknown [29]. The deletion of *ocd* in an *argF*^-^*, argR*^-^ strain of *C. glutamicum* increased ornithine production by this strain. Supplementing 5 mM proline improved ornithine production further, which was hypothesized to indicate a possible role of OCD in the conversion of proline to ornithine [30]. As shown here, overexpression of *ocd* from *C. glutamicum* neither entailed proline production nor detectable OCD activity, although a SDS-PAGE of crude extracts revealed overproduction of the protein. Thus, the protein encoded by *ocd*_*Cg*_ either does not possess OCD activity or its activity was too low to be detected. It is interesting to note that multiple protein sequence alignments of biochemically characterised OCDs with putative OCDs [24,31] revealed that some conserved active site residues of OCDs are not conserved in OCD_*Cg*_. Instead of Arg45 (numbering according to OCD from *P. putida*), one of three residues whose side chains interact with the ornithine carboxyl group, OCD_*Cg*_ contains a glutamate residue and Asp228, whose side chain forms a hydrogen bond with the leaving ammonia group, is a glycine residue in OCD_*Cg*_. The lack of conservation of these and further amino acids might explain why no OCD activity could be detected in *C. glutamicum*. Physiologically, *C. glutamicum* differs from pseudomonads that typically are able to catabolise arginine and ornithine as sole carbon and nitrogen source. In most *Pseudomonas* species utilisation of ornithine as carbon source involves succinylation of ornithine, however *P. putida*, which is devoid of such activity, catabolises ornithine via OCD and subsequently via proline degradation [32]. OCD is also involved in opine degradation by *Agrobacterium tumefaciens* with e.g. the nopaline catabolism region of Ti plasmid C58 encoding OCD for degradation of nopaline via arginine and ornithine to proline [33]. The observation that *C. glutamicum* does not appear to be able to utilise proline, as shown in this study, or ornithine (unpublished observation) as sole nitrogen or carbon source is commensurate with the lack of OCD activity. It remains to be shown if the protein annotated as putative OCD is active as ornithine cyclodeaminase, or whether it catalyses another reaction.

Heterologous expression of *ocd*_*Pp*_ by the ORN1 strain resulted in proline accumulation, and a significant increase in production could be achieved by changing the stop codon from TGA to TAA. While examples of modulating translation initiation by changing the start codon or by changing the sequence or spacing of the ribosome binding site exists for *C. glutamicum*[23], modulating translation termination by altering the stop codon has to the best of our knowledge not yet been reported. Increased OCD levels and activities as consequence of changing the stop codon from TGA to TAA is in line with a bioinformatic study on codon usage of *C. glutamicum*. Putative highly expressed genes exhibited a strong bias for the UAA stop codon, while such a preference was not observed in lowly expressed genes [34]. It is likely that optimisation of sense codons of *ocd*_*Pp*_ to fit the sense codon preference of *C. glutamicum* better, could contribute to a further increase in proline production.

As previously demonstrated, glutamate is not limiting the flux through the ornithine pathway, rather it is the feedback inhibition of NAGK by arginine and potentially the feedback inhibition of OAT by ornithine [18,21]. Accordingly, overproduction of feedback-alleviated NAGK improved proline production. In addition, growth was affected as less biomass formed and as the growth rate was reduced. It is noteworthy that proline production already started early during growth which may be beneficial for the overall space-time yield of the process. Moreover, formation of the by-products valine and threonine that are not amino acids of the glutamate family was reduced. As trace amounts of glutamate could be detected in the samples taken for all strains constructed (JJ001-JJ006), this is an indication of that the bottleneck in proline production is located between glutamate and proline. A further improvement of the conversion of glutamate to ornithine can be envisioned by alleviating a potential feedback inhibition of OAT and/or employing a bifunctional enzyme with OAT and NAGS activities.

## Conclusions

Heterologous overexpression of *ocd* in *C. glutamicum* ORN1 resulted in the overproduction of proline through the ornithine pathway. *C. glutamicum* JJ004 had a yield of 0.31 ± 0.01 g proline/g glucose. Alleviating feedback inhibition of N-acetylglutamate kinase entailed growth-coupled and improved proline production with a yield of 0.36 ± 0.01 g/g. The addition of proline to the product palette of the ornithine producing strain ORN1 emphasises that this strain might be exploited as platform strain for industrially relevant bioproducts such as ornithine, proline, putrescine, spermidine, citrulline, and arginine. Moreover, engineering strategies of the platform strain can easily be transferred and applied to improve derived producer strains.

## Methods

### Strains, plasmids, and media

*C. glutamicum* strain ATCC 13032 [35], ORN1 [21], and its derivatives have been used in this study. As ornithine is a precursor of several interesting products, such as amino acids, di- and polyamines of the glutamate family, ORN1 has the potential to serve as a platform strain. *E. coli* DH5α [36] was used for the cloning procedures and cultured at 37°C in Lysogeny Broth (LB) [37] or on LB-agar. Competent *E. coli* cells and molecular techniques were performed according to standard procedures [37]. Chromosomal DNA from *C. glutamicum* and *Pseudomonas putida* KT2440 was isolated by resuspending overnight cultures in 360 μL 50 mM Tris–HCl (pH 8) followed by the addition of a spatula tip of lysozyme, and incubation at 37°C for two hours. Thereafter the procedure “DNA purification from tissues” with the QiaAmp DNA mini kit (Qiagen, Hilden, Germany) was followed. Preparation and transformation of *C. glutamicum* competent cells was performed according to published methods [38]. Plasmids pVWEx1-*ocd*_*Pp*2_ and pVWEx1-*ocd*_*Pp*1_ were constructed by amplifying *ocd* from *P. putida* [NCBI-GeneID: 1046312] with primers *ocd2*-FW (CTTctgcagAAGGAGATATAGATATGACGTATTTCATTGATGTTCCA) and *ocd3*-RV (CCTggtaccTTAGGCAACCCGTCGGATAC, the stop codon was modified from TGA to TAA) or *ocd2*-RV (CCTggtaccTCAGGCAACCCGTCGGATAC). The amplified fragments were treated with *Kpn*I and *Pst*I and ligated with similarly treated pVWEx1. Plasmid pVWEx1-*ocd*_*Cg*_ was constructed similarly, however primers *ocd1*-FW (CTTctgcagAAGGAGATATAGATATGACCGCAACCTACACCACTG) and *ocd1*-RV (CCTggtaccTCAAGCCAGTGCGGGTG) were used for the amplification of *ocd* from *C. glutamicum* [NCBI-GeneID: 3343467]. The construction of pEKEx3-*argB*_A49VM54V_ has been described elsewhere [20]. Plasmids pVWEx1, pVWEx1-*ocd*_*Cg*_, pVWEx1-*ocd*_*Pp*2_, pVWEx1-*ocd*_*Pp*1_, pEKEx3, and pEKEx3-*argB*_A49VM54V_ were transformed into ORN1 resulting in strains JJ001, JJ002, JJ003, JJ004, JJ005, and JJ006, respectively. Brain heart infusion broth (BHI, Roth Chemie GmbH, Karlsruhe, Germany) was used for inoculation of precultures, while CGXII minimal medium 40 g/L glucose or CGXIIm (CGXII but without ammonium sulfate) 35 g/L glucose was used for growth and proline production.

### Culture conditions

*C. glutamicum* was inoculated to an OD of 1 in 50 mL minimal medium, 0.75 mM arginine, 1 mM Isopropyl-β-D-thiogalactopyranosid (IPTG), 25 μg/mL kanamycin, and when required 50 μg/mL spectinomycin on a rotary shaker (120 rpm) in baffled shake flasks at 30°C. Cultivations were always performed in triplicates. Growth was monitored measuring the OD at 600 nm using a spectrophotometer (V-1200, VWR, Radnor, PA, USA). 5 g/L urea and N-equimolar concentrations of ammonium sulfate and ammonium chloride were used for screening of nitrogen sources. For the screening of nitrogen sources and the Plackett-Burman design, cells were grown in 48-well flower plates using the Biolector microfermentation system (m2p-labs GmbH, Aachen, Germany). 1 mL medium was used per well with a shaking frequency of 1100 rpm. Biomass formation was measured as backscattered light intensity sent at 620 nm with a signal gain factor of 20.

### Utilisation of proline as carbon and nitrogen source

*C. glutamicum* was inoculated to an OD of 1 in variants of CGXII medium. For the utilisation of proline as carbon source CGXII with 20 g/L glucose or with a C-equimolar concentration of proline was used. For the utilization of proline as nitrogen source CGXII with 5 g/L urea and 20 g/L ammonium sulfate, with a N-equimolar concentration of proline, or with no nitrogen source added, was used.

### Screening of medium components using the Plackett-Burman design

A Plackett-Burman design [39] for 19 factors (including seven dummies) with 20 runs was employed to screen for factors that significantly affect proline production through the ornithine biosynthetic pathway. The dummies serve as a measure for the error in estimating the main effects. The medium components were screened at a low (−) and a high (+) level, where the concentrations can be found in Table 6 and the design in Table 4. The concentrations of the two levels were selected based on a literature search and preliminary results. The effect of each factor on proline production was determined by the equation:

$$E\left(X_{i}\right)=2\left(\Sigma Y_{+i}–\Sigma Y_{-i}\right)/N$$

where *E*(*X*_*i*_) is the factor main effect, *Y*_+*i*_ and *Y*_-*i*_ are the proline concentrations in which the factors being tested are at their high and low levels respectively, N is the number of runs. The *t*-values of the factor main effects were plotted in a Pareto chart, and evaluated based on a *t*-value and a Bonferroni limit [40]. The t-test assesses the risk of declaring an effect significant, when it actually was caused by chance. The Bonferroni correction is more conservative taking the number of estimated effects into account by division by the desired probability for the risk value. Effects above the Bonferroni limit are likely not stochastic [40]. The experiment was designed, and obtained data analysed, using the software Design-Expert 8.0.7.1 (Stat-Ease Inc., Minneapolis, USA). The experiment was carried out in duplicates, where the mean was considered the response (Table 4).

**Table 6:** Concentration levels used in the Plackett-Burman experiment

**Factor**	**Name**	**Level**	
		**-**	**+**
A (g/L)	Glucose	20	40
B (g/L)	Urea	2.5	10
C (g/L)	KH_2_PO_4_	0.5	2.0
D (g/L)	K_2_HPO_4_	0.5	2.0
E (g/L)	CaCl_2_	5.0·10^-3^	2.0·10^-2^
F (g/L)	MgSO_4_	1.3·10^-1^	5.0·10^-1^
G (g/L)	MOPS	31	52
H (mL/L)	Trace metals	7.5·10^-1^	2.0
I (g/L)	Biotin	1.0·10^-4^	4.0·10^-4^
J (g/L)	Prochatechuic acid	1.5·10^-2^	6.0·10^-1^
K (g/L)	IPTG	1.2·10^-1^	3.6·10^-1^
L (g/L)	Arginine	1.3·10^-1^	1.7·10^-1^
D1-D7	Dummy	0	0

### Ornithine cyclodeaminase assay

BHI broth supplemented with 25 μg/mL kanamycin and 1 mM IPTG was inoculated to an OD of 1 and grown for 4 h at 30°C. Cells were harvested and washed in 20 mM KH_2_PO_4_ (pH 8.2). Then, cells were lysed by means of sonication (Ultraschalldesintegrator Sonoplus GM 200, Sonotrode M72, Bandelin electronic GmbH & Co KG, Berlin, Germany) for 6 min (cycle 0.5, amplitude 55) and centrifuged for 60 min at 4°C and 14600 rpm.

Crude extracts were purified using PD10 desalting columns (GE Healthcare, Chalfont St Giles, United Kingdom) with 20 mM KH_2_PO_4_ (pH 8.2). The reaction solution consisted of 20 mM KH_2_PO_4_ (pH 8.2) and 0.5 mM NAD^+^. Tubes with reaction solution and between 0.08 and 0.4 mg protein per 250 μL reaction were equilibrated to 30°C for 3 min in a water bath. The reaction was initiated upon the addition of 25 mM L-ornithine. The reaction was stopped upon addition of 50% formic acid. The samples were then neutralized with 10 N KOH and precipitate was pelleted by centrifugation. Reactions were performed in triplicates with two enzyme concentrations. The conversion of ornithine to proline was measured by HPLC. Unpurified extracts were analysed by SDS-PAGE, and protein quantification was performed by the procedure of Bradford with bovine serum albumin as the standard [41].

### N-acetylglutamate kinase assay

Crude extracts were prepared as stated for the ornithine cyclodeaminase assay. The NAGK activity assay was performed as described by Haas and Leisinger [42]. The assay was performed in triplicates and carried out at 30°C at pH 7.2. One enzyme unit is the amount of enzyme that catalyses the formation of 1 μmol of product in 1 min.

### Amino acid and glucose determination

Extracellular amino acids and carbohydrates were quantified by means of high-pressure liquid chromatography (1200 series, Agilent Technologies Deutschland GmbH, Böblingen, Germany). Samples were withdrawn from cultures, centrifuged (13,000 × *g*, 10 min), and the supernatant was used for analysis. For the detection of amino acids, samples were derivatised with 9-fluorenylmethyl chloroformate (FMOC) or *ortho*-phthaldialdehyde, separated on a system consisting of a pre-column (LiChrospher 100 RP18 EC-5μ (40 × 4 mm), CS-Chromatographie Service GmbH, Langerwehe, Germany) and a main column (LiChrospher 100 RP18 EC-5μ (125 × 4 mm), CS-Chromatographie), and detected with a fluorescence detector (FLD G1321A, 1200 series, Agilent Technologies). L-Asparagine was used as internal standard. For the detection of carbohydrates the separation of the analyte was achieved with a column for organic acids (300 × 8 mm, 10 μm particle size, 25 Å pore diameter, CS-Chromatographie) and a refractive index detector (RID G1362A, 1200 series, Agilent Technologies) was used. Derivatisation and quantification was carried out according to published methods [22] with the following modifications of the quantification of FMOC derivatised samples: The mobile phases used were A: 50 mM sodium acetate (pH 4.2) and B: acetonitrile. The gradient used was: 0 min 38% B, 5 min 38% B, 12 min 57% B, 14 min 76% B, 15 min 76% B, and 18 min 38% B.

## Competing interests

The authors declare that they have no competing interests.

## Authors’ contributions

VFW and JVKJ designed the experiments. JVKJ conducted the experiments, analysed the results, and wrote the manuscript. VFW reviewed and revised the manuscript. All authors read and approved the final manuscript.
